# Pseudotumor Cerebri and Glymphatic Dysfunction

**DOI:** 10.3389/fneur.2017.00734

**Published:** 2018-01-16

**Authors:** Marcio Luciano de Souza Bezerra, Ana Carolina Andorinho de Freitas Ferreira, Ricardo de Oliveira-Souza

**Affiliations:** ^1^Rio Sono Clinic, Rio de Janeiro, Brazil; ^2^Instituto D’Or de Pesquisa e Ensino (IDOR), Rio de Janeiro, Brazil

**Keywords:** pseudotumor cerebri, idiopathic intracranial hypertension, glymphatic system, paravascular spaces, aquaporin-4, cerebrospinal fluid, glymphatic clearance

## Abstract

In contrast to virtually all organ systems of the body, the central nervous system was until recently believed to be devoid of a lymphatic system. The demonstration of a complex system of paravascular channels formed by the endfeet of astroglial cells ultimately draining into the venous sinuses has radically changed this idea. The system is subsidized by the recirculation of cerebrospinal fluid (CSF) through the brain parenchyma along paravascular spaces (PVSs) and by exchanges with the interstitial fluid (IF). Aquaporin-4 channels are the chief transporters of water through these compartments. This article hypothesizes that glymphatic dysfunction is a major pathogenetic mechanism underpinning idiopathic intracranial hypertension (IIH). The rationale for the hypothesis springs from MRI studies, which have shown many signs related to IIH without evidence of overproduction of CSF. We propose that diffuse retention of IF is a direct consequence of an imbalance of glymphatic flow. This imbalance, in turn, may result from an augmented flow from the arterial PVS into the IF, by impaired outflow of the IF into the paravenous spaces, or both. Our hypothesis is supported by the facts that (i) visual loss, one of the main complications of IIH, is secondary to the impaired drainage of the optic nerve, a nerve richly surrounded by water channels and with a long extracranial course in its meningeal sheath; (ii) there is a high association between IIH and obesity, a condition related to paravascular inflammation and lymphatic disturbance, and (iii) glymphatic dysfunction has been related to the deposition of β-amyloid in Alzheimer’s disease. We conclude that the concept of glymphatic dysfunction provides a new perspective for understanding the pathophysiology of IIH; it may likewise entice the development of novel therapeutic approaches aiming at enhancing the flow between the CSF, the glymphatic system, and the dural sinuses.

## Introduction

### A Brief Overview Of The Glymphatic System

Recent investigations in experimental animals have shown that, from a rheological standpoint, the cerebrospinal fluid (CSF) resembles the peripheral lymphatic system in critical ways. Hints on the existence of this system came from the identification of endothelial lymphatic cells by intraventricular injections of immunohistochemical markers in meningeal vessels. These vessels drain the CSF and contain T and MHCII^+^ cells (1). Additional demonstration of the role of the astroglia has led researchers to coin a new term for this cerebral lymphatic system, the “glymphatic system” (2). Because the glymphatic system develops earlier than the arachnoid villi, it represents a critical outflow system for the CSF during fetal life up to the end of the neonatal period (3, 4).

The discovery of the glymphatic system has challenged classical views on the “third circulation” and rapidly galvanized the interest of basic researchers. Over the past few years, the recognition that the glymphatic system may be involved in the pathophysiology of common neurologic diseases, such as idiopathic intracranial hypertension (IIH), has also attracted the interest of clinicians. These advances require a review of some critical aspects of some of the classical views on CSF circulation.

### Changes of the Classical View on the Circulation of the CSF by the Glymphatic Concept

According to classical knowledge, the CSF is formed as an ultrafiltrate of the blood in the choroid plexus at a daily rate of 500 mL, but the CSF volume in the human central nervous system (CNS) is 150 mL, indicating the CSF is replaced three to four times a day (5, 6). From the ventricles, the CSF passes through the foramina of Luschka and Magendie out of the ventricular system into the subarachnoid space (SAS), whence it either flows downward, around the spinal cord, or upward, over the cerebral convexities (7). From this compartment, the CSF is reabsorbed by the arachnoid granulations of the dural sinuses or drained from the cribriform plate into the cervical lymphatic system (2, 8).

**Figure 1:**
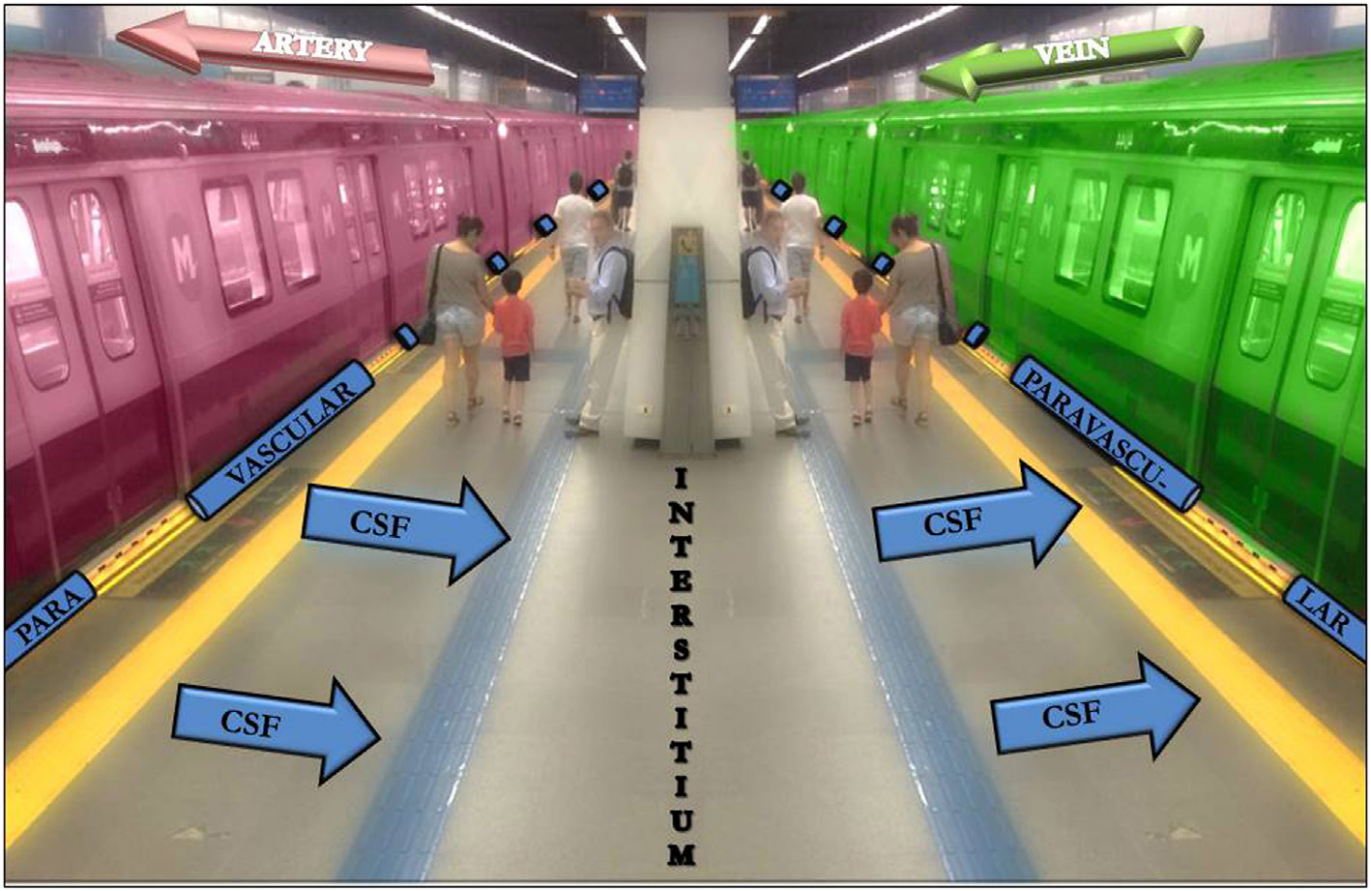
The subway platform as an analogy to the cerebrospinal fluid (CSF)-interstitium exchange. From the subarachnoid space, the CSF gets into the arterial paravascular space (left gap between the red train and the platform). Subsequently, the CSF is transported into the brain parenchyma (the platform). The CSF movement into the parenchyma drives convective interstitium fluid toward the paravenous spaces (right gap between the green train and the platform) surrounding the deep veins. People that cross the platform may be compared with different solutes.

The classical model has been modified in critical points by the results of tracer studies that have shown that a large proportion of the CSF recirculates through the brain parenchyma along paravascular spaces (PVSs) with direct communication with the interstitial fluid (IF) all over the way. The CSF also drains into the lymphatic system through the meningeal sheaths of the spinal and cranial nerves, especially the optic, trigeminal, facial, and vestibulocochlear (2, 3). The interstitial solutes are then collected along the paravenous spaces (Figure 1) and gain access into the sinus-associated lymphatics either directly, since these large veins merge into the dural sinuses, or indirectly, *via* the cisternal CSF. In this sense, the paravascular pathways within the CNS parenchyma enable the clearance of solutes from the brain to the CSF, whereas the extra-axial meningeal lymphatic vessels propel the solute-laden CSF on to the systemic vascular system (2, 4, 8–10).

The role of the PVS has been documented in the optic nerves. Denniston et al. (11) argue that local inflammation results in increased vascular permeability that leads to paravascular enlargement, which is visible as “black holes” in the papilledema of raised intracranial pressure (ICP).

Injections of paramagnetic agents and fluorescent tracers in the SAS of mice have shown that the CSF moves swiftly from the SAS into the Virchow–Robin PVSs (2, 8). Lining these arteries and arterioles are contiguous astrocytic endfeet, the “glia limitans,” which provide a high-resistance barrier between the paravascular and interstitial compartments. The density of the water channels of the astrocyte endfeet is comparable to that of the kidney, an organ specialized in water transport (7). The glial endfeet contain 10-fold higher density of aquaporin-4 (AQP4) than non-endfeet membranes. The glia limitans curbs the access of large molecules from the PVS into the interstitium. However, roughly 50% of the surface area of the capillary-facing endfeet is occupied by AQP4. The AQP4 channels mediate transglial movement of water between the paravascular and interstitial compartments, thus allowing the transportation of CSF from the Virchow–Robin spaces into the cerebral IF (12). This microscopic CSF flow removes solutes, amyloid, and other waste products, exiting the IF again through the AQP4 channels, thus bypassing the arachnoid pathways of detritus removal (8).

The movement of CSF into the brain *via* the Virchow–Robin spaces is enhanced by arterial pulsations and sleep (3). In mice, sleep fosters a 60% increase of the brain’s interstitial space. This increase results from an expansion of the intercellular volume, which helps pushing fluid through the brain tissue (1, 13). Jessen et al. (14) attributed this change in intercellular volume to the regulator role executed by norepinephrine during wakefulness. The use of norepinephrine receptor antagonists in awake mice resulted in an increase in interstitial space almost comparable to that observed during sleep or anesthesia (13). The burst release of norepinephrine during arousal increases the cellular volume resulting in a decrease in the interstitial space. On the other hand, sleep increases interstitial volume and reduces tissue resistance toward convective flow, thus permitting a freer CSF–ISF exchange (15).

Recently, the importance of the glymphatic system during sleep and anesthesia was confirmed by experimental manipulations that impair glymphatic clearance. These manipulations included the following: (i) administration of acetazolamide, a carbonic anhydrase inhibitor that reduces CSF production, (ii) cisterna magna puncture, which eliminates the small pressure gradients that allow the convective exchange of CSF with the IF, (iii) AQP4 deletion, and (iv) body position adjustment (16). In all these conditions, the expected decline in tissue lactate during sleep did not occur.

The glymphatic system has also been implicated in the removal of heat excess from the brain. Narebski stipulated a brain–blood temperature gradient in which the Circle of Willis is approximately 0.5°C cooler than the brain tissue, which is under metabolic heat. The cooler blood flow was defined as the main mechanism for the removal of heat excess by convection (17). Refreshing of cortical vessels, in turn, is guaranteed by their course in the SAS, which allows CSF-venous thermal exchange. Moreover, the close interaction between CSF and blood occurs at the capillary level through the PVS and the interstitial space (18).

In the following section, we review the clinical features of IIH, a common neurological disorder that may be caused by glymphatic dysfunction.

### IIH: A Selected Clinical and Pathophysiological Overview

The expressions “pseudotumor cerebri” and “benign intracranial hypertension” were originally applied to patients with increased ICP who followed an apparently benign course in the absence of an expanding mass. Subsequent studies have shown that high ICP is not always benign or unrelated to a “false” tumor; therefore, its terminology was changed to IIH (19). The prevalence of IIH in the general population is 0.5–2/100,000 but increases to 12–20/100,000 in obese women of childbearing age. There is a 3:1 to 18:1 female predominance, and obesity is observed in 59–100% of cases (20). According to the modified Dandy criteria, IIH is defined by an increase in ICP leading to headache, papilledema, visual symptoms, pulsatile tinnitus, and vomiting; lateralizing signs, except for bilateral sixth nerve palsy, are not part of the clinical picture; no cause for the increased ICP is apparent on MRI scans; the CSF opening pressure is higher than 25 cm H_2_O, and the CSF composition is normal (20). Sixth nerve palsy occurs in approximately 12% of adult patients (21). There are some intriguing clinical differences regarding the visual outcome in older patients and men with IIH. Patients older than 50 years have fewer complaints of headache and visual disturbances, and the visual prognosis is better in the elderly. By contrast, the visual function of men with IIH needs to be followed-up closely because they may not report other IIH symptoms, such as headache (22).

Although IIH has been recognized for over a century, its pathogenesis remains unclear (19, 20). Several mechanisms have been proposed, such as parenchymal edema, increased cerebral blood volume, CSF overproduction, venous outflow obstruction, and compromised CSF resorption (23). In recent years, a possible contribution of inflammation has been invoked as another possible mechanism (19). Despite intensive study on the pathogenesis of IIH, no single explanation has so far provided a comprehensive answer for the exact cause of IIH (19).

### Neuroimaging Findings in IIH

Sahs and Joynt reported evidence of intracellular and extracellular edema in IIH (24). However, subsequent MRI studies have disputed the increase in water content of the brain and water diffusion in the subcortical white matter of IIH patients (25–28).

“Empty sella” refers to an MRI sign whereby the pituitary gland appears compressed against the sella turcica assuming a concave crescent shape. An empty sella is usually a consequence of increased ICP, which leads to herniation of the SAS through the sella diaphragm. Empty sella has been associated with IIH, with prevalence rates that range from 2.5%, for a total empty sella, up to 94%, for a partial empty sella. This could be in part due to a lack of strict anatomical criteria for what constitutes an empty sella in the first place. Enlargement of the sellar area provided high sensitivity (100%) and specificity (90%) in differentiating IIH from normal controls. The area of the pituitary has been reported to be very sensitive (100%) for detecting improvement following pharmacological treatment of IIH. Furthermore, an empty sella is a non-specific finding, which is often incidentally found in healthy subjects as well as in obesity, meningioma, pediatric nevoid basal cell carcinoma, and following therapy for growth hormone deficiency. Regardless of its limited specificity, the finding of an empty sella in conjunction with other radiological signs support the diagnosis of IIH provided tumor, venous thrombosis, and infection are ruled out (29).

Chronic increased ICP leads to bone erosion and remodeling, resulting in widening of the bone canals of the cranial base. Enlargement of the foramen ovale has high specificity (81%), but low sensitivity (50%) for a diagnosis of IIH. The jugular foramen and hypoglossal canal were also reported to be enlarged in IIH. Enlargement of CSF spaces or spontaneous CSF-filled outpouchings of the dura (meningoceles) resulting from chronic increased ICP may also support the diagnosis of IIH. These enlargements have been reported in Meckel’s cave, the oculomotor nerve sheath, and in the CSF spaces around the abducens nerve (29).

Neuroimaging is decisive for ruling out causes of raised ICP in space-occupying lesions. Venous sinus thrombosis can present with severe headache from intracranial hypertension secondary to impairment of CSF reabsorption, which can mimic IIH symptoms (29). MRI of the head and orbit with intravenous contrast and magnetic resonance venography are the modalities of choice to exclude structural lesions prior to an IIH diagnosis (30).

Intraorbital imaging shows dilated optic nerve sheathes, posterior globe flattening, and protrusion of the optic nerve head. Transorbital ultrasonography is a reliable and non-invasive imaging technique to show enlargement of the optic nerve sheath diameter (ONSD) with good sensitivity (90%) and specificity (84%) at a cutoff value of 5.8 mm in detection of IIH. The ONSD of 5.5 mm predicted a CSF pressure of 20 cm H_2_O with 88–100% sensitivity and 93–100% specificity. Optic disc elevation can also be detected by ultrasound. MRI is another reliable imaging modality for the detection of increased ONSD with a sensitivity of 72–80% and specificity of 96%. ONSD decreases 30 min after lumbar puncture (LP), while normalization of optic disc protrusion requires longer treatment times. Flattening of the posterior globe in MRI is highly specific (close to 100%), but not sensitive, to IIH. Although these imaging features reliably differentiate IIH patients from individuals without intracranial hypertension, these signs can also be seen in increased ICP from other causes, as in cerebral venous thrombosis (29).

Stenosis at some point of the dural sinus system has been reported in 30–93% of IIH patients. Stenosis of the transverse and sigmoid sinuses detected by magnetic resonance venography is reliable markers of IIH with high sensitivity and specificity (93% for both). It is still debated whether venous sinus stenosis plays a causal role or results from increased ICP. Acute CSF volume reduction by CSF diversion or a single LP can ameliorate the symptoms of venous sinus stenosis. Stenting of transverse sinus stenosis, in turn, can improve intracranial hypertension. These findings suggest that venous sinus stenosis and intracranial hypertension share a circular loop relationship (29).

### MRI Assessment of Glymphatic Function and Dysfunction

In contrast to the blood, which runs through a network of dedicated vessels, the CSF flows through paravascular channels and brain parenchyma at a much slower pace (31). The development of techniques to study the flow patterns of glymphatic transport through the brain *in vivo* is an urgent need so that the role of glymphatic dysfunction in the development of symptoms in several common pathological states can be better understood.

3D-FLAIR (hT2-FL) sequences are sensitive to very low concentrations of gadolinium (IV-GBCA), which appears as a slight enhancement of CSF 4 h after a single dose of IV-GBCA in healthy subjects. Following this lead, Naganawa et al. (32) investigated the possibility of enhancing the PVS as well. As a result, some of the PVS signals were visually more intense than that of the CSF 4 h after IV-GBCA. The authors argued that simple penetration of the CSF into the IF of the PVS was not sufficient to explain the increased PVS signal. They then suggested that the PVS has an active mechanism such as the absorption of water in the PVS or the secretion of gadolinium into the PVS (32).

The human glymphatic system was successfully imaged with T_1_-weighted MRI in a recent investigation with the intrathecal contrast agent gadobutrol injected *via* the lumbar route (33). There was a persistent enhancement of the brain parenchyma in patients with IIH, which the authors interpreted as a direct evidence for impaired glymphatic clearance. This is the first study to provide unequivocal evidence on the feasibility of imaging the human glymphatic system in IIH *in vivo*.

## Hypotheses

The main hypotheses of this article may be summarized as follows:

1. Glymphatic dysfunction compromises the clearance of IF and leads to diffuse stagnation of flow and solutes in (a) the interstitium, (b) in the PVSs, or (c) in both, resulting in IIH; and
2. Glymphatic dysfunction results from (a) augmented flow from arterial PVS to the IF, (b) impaired outflow of IF into the paravenous space, or (c) both.
3. Hypotheses (1) and (2) are not mutually exclusive and may assume different weights in individual patients.

## Evaluation of the Hypotheses

### Evidence for Glymphatic Dysfunction

Kress et al. (34) observed a reduction of 40% of β-amyloid (Aβ) clearance in the brain of aged in comparison with young mice. They also noted a 27% decrease in arterial wall pulsatility in the aged mice, which is an important contributor to paravascular flow along the glymphatic pathways (34). Vessel stiffness results in decreased arterial pulsation and a reduction in IF flow (32). As shown by Kyrtsos and Baras, an increase in vessel stiffness from baseline by a factor of just 2 leads to a significant increase in the deposition of Aβ in brain parenchyma (35).

*In vitro* study showed that inducing microglia to express macrophage like surface proteins was especially effective at lowering lysosomal pH and activating the degradation of fAβ sheet structures (36). In agreement with this result, it was suggested that proton pump inhibitors (PPIs) would lead to the enrichment of Aβ in cell model by inhibiting acidification of lysosomes. PPIs have inhibitory properties at vacuolar-type H^+^-adenosine triphosphatase. As a result, PPIs may contribute to the inhibition of acidification and reduce Aβ degradation (37). After the proper degradation, it would be on account of glymphatic system to promote the wash out of the derived metabolites.

The loss of astroglial AQP4 polarization has also been associated with an impairment of CSF-IF exchange, which may underpin the slower CSF clearance and increased protein content of the aging brain (1). Zeppenfeld et al. (38) evaluated the expression and localization of AQP4 among 79 samples of postmortem brain tissue from participants with a clinical history of Alzheimer disease (AD) or no neurological disease (controls). The authors observed that AQP4 expression increased with age in both groups. However, the loss of AQP4 in astrocytic paravascular endfeet was noted in individuals with AD, and this loss was proportional to neurofibrillary and Aβ pathology independently of age (38).

Recently, Taoka et al. (39) attempted to demonstrate CSF-IF exchange with diffusion tensor imaging along the perivascular spaces. They focused on the body of the lateral ventricles, where medullary veins and PVS are perpendicular to each other and run in the right–left direction (*x*-axis). Projection fibers follow a head-to-foot direction (*z*-axis) and the intra-hemispheric fascicles run in the anterior–posterior direction (*y*-axis). This configuration—in which vessels and white matter are perpendicular to each other—allowed an independent analysis of diffusivity in PVS, which was taken as a surrogate of glymphatic function. The authors found a significant positive correlation between PVS diffusivity and Mini-Mental State Exam scores, indicating impaired water diffusivity in the PVS of projection and association fibers, and this impairment was related to AD severity. The authors concluded that glymphatic dysfunction is the rule in patients with severe AD. The results were consistent with the impaired glymphatic activity observed in experimental animals (39).

### Epidemiological, Clinical, and Radiological Conditions Related to Intracranial Hypertension

Obesity is present in up to 100% of patients with IIH. Obesity has increasingly been perceived as an inflammatory disorder (20). A study comparing high-fat diet between obesity-prone and obesity-resistant mice disclosed that obesity and not diet was the determinant of lymphatic dysfunction. This disturbance would be bound by a triggered perivascular inflammation (40). In addition, markers of inflammation were found in CSF of IIH patients, with the concentration of CC chemokine ligand 2 (CCL2) being significantly higher than in controls (19). As intercellular effectors, CCL2, released specifically by astrocytes, are important recruiters of perivascular leukocytes in CNS autoimmune inflammation (41). From this, it might be speculated the role of inflammation in obesity induced in the increase of BBB permeability. It would lead to higher volume of the content in PVS and interstitium, causing an overload to the glymphatic system.

The main morbidity of IIH is visual loss: transient obscurations occur in roughly 70% of patients, and visual loss or blurring in 32% (20). The role of impaired glymphatic functioning behind eye involvement is indirectly shown by the fact that the optic nerve runs a long extracranial course in its meningeal sheath, a factor that may predispose to drainage difficulty. Accordingly, IV-GBCA enhancement occurred earlier in the optic nerve than in the CSF of the ambient cistern in humans, which presupposes that at least the content of CSF from vascular contribution—probed by gadolinium infusion—flows from the cranial nerve or nerve sheath to the CSF (1, 3, 32).

The AQP4 channels mediate the astroglial water flow that couple para-arterial CSF influx and paravenous IF clearance. It is highlighted by the image study set among mice, where AQP4-null mice had 70% of reduction in clearance of radiolabeled [3H]mannitol (2). Since AQP4 is expressed at multiple sites in the CNS, including many regions from retinal astrocytes to perinodal axolemma of optic nerve, we may speculate on the possible visual damage associated with intracranial hypertension if IIH was bounded to AQP4 deficiency (42).

Another frequent symptom of IIH is tinnitus, present in 52–60% of patients (20). This finding could reflect glymphatic dysfunction of the vestibulocochlear nerve, whose sheaths are also drained by the lymphatic system. Besides, the cochlear duct establishes a communication between the SAS of the posterior cranial fossa and the perilymphatic space of the cochlea (3). This communication allows the reverberation of ICP variations over cochlear function.

Beyond the role of AQP4 as doormen, a study in mice verified that the PVS cross-sectional area around veins tends to be smaller. This finding speaks for a natural difficulty to CFS outflow from brain compared with its inflow. The same experiment showed that cortical spreading depression, which is a trigger to constriction–dilation–constriction in arteries, induced the closure of the PVS. It happened not only in the para-arterial but also in the paravenous space, despite the absence of changes in venous diameter (43). It is thus possible that the para-arterial exerts a control function on the paravenous spaces. It further indicates that both PVS share the same efferent closure mechanisms, which is consistent with the AQP4 functional role. Therefore, in an opposite situation, the dilatation of para-arterial space might be followed by dilatation of paravenous space. In this case, if a dilatation leads to augmented influx to IF, it would be reasonable that the outflow would also be facilitated. However, since the para-arterial is larger than the paravenous space, the content that gets in is greater than the content that gets out of the IF. In short, both shrinking and widening of PVS may contribute to CSF stagnation. Widening is a possible scenario taking in account the inflammation generated by obesity, which is present on most of IIH patients. Increased expression of AQP4 has been found in inflammatory conditions, including bacterial meningitis and inflammation secondary to intraparenchymal injection of lipopolysaccharides (11).

Schain et al. also showed that the cortical spreading depression-induced parenchymal swelling has the potential to spread from cortical layer I to the pia (43). The swelling may have a domino effect promoting a vicious cycle that makes the passage of IF content to paravenous space even harder.

### Normal-Pressure Hydrocephalus

Following an inverse line of thought, when ventriculoperitoneal shunting to drain CSF is performed, patients with idiopathic normal-pressure hydrocephalus (iNPH) may exhibit remarkable improvements, especially if the symptom presented is gait impairment (44). iNPH is a disease typically seen in the elderly and is characterized by enlargement of ventricles, normal CSF (9, 43, 44). Clinically, NPH is described by the insidious onset of the triad: dementia, gait disturbance, and urinary incontinence (44, 45). Once MRI findings are suggestive of iNPH, CSF tap test—the removal of 30–60 mL of fluid—can indicate shunt outcome. Clinical improvement after the procedure is generally a positive predictor of shunting. Even though there is no improvement in CSF tap testing, external lumbar drainage, for 72 h, is indicated and works as a better predictor of treatment (44). We speculate that restoring fluid flows through the glymphatic system might mediate the resolution of symptoms in these patients. Bradley (46) has argued that patients with iNPH may have had benign external hydrocephalus in infancy, which was compensated by a pathway that allowed the CSF to exit through the glymphatic system. In late adulthood, white matter ischemia and the increased pathological affinity between the bare myelin protein and the CSF increases resistance to the extracellular outflow of CSF exposing the latent hydrocephalus (46).

The impairment in dynamic of CSF flow in iNPH is illustrated by the study of Jeppsson et al. (47) where it was found low levels of all the amyloid precursor protein (APP) fragments (Ab38, Ab40, Ab42, sAPPa, and sAPPb) in the CSF of patients. After shunting, all the proteins (except t-tau) increased in the ventricle CSF (47). Graff-Radford (48) defends that when the patient has a shunt placed, it is generated room for the IF space increase during sleep and provide better drain into the CSF.

### Glaucoma

Wostyn et al. (49) discuss the hypothesis of glymphatic dysfunction and Aβ being involved in glaucoma neurotoxicity. In high-tension glaucoma, retinal ganglion cells (RGC) of rat subjected to chronic elevation of intraocular pressure (IOP) exhibit caspase-3-mediated abnormal processing of APP with increased expression of Aβ. Aβ, in turn, may be implicated in the development of RGC apoptosis. In other respects, normal-tension glaucoma would stem from glymphatic pathway dysfunction that would hamper the clearance of Aβ from the optic nerve. Therefore, in normal-tension glaucoma, abnormal clearance of Aβ from the optic nerve may predominate as a result of glymphatic pathway dysfunction while IOP-induced Aβ generation may overweight in high-tension glaucoma (49).

There is growing evidence of lower ICP in patients with primary open angle glaucoma (POAG) when compared with non-glaucomatous control subjects and additionally, is lower in the normal-tension versus the high-tension form of POAG. The authors sustain that the lower ICP reported in POAG patients could be an indicator of decreased CSF production and turnover, what may demonstrate that changes in CSF circulatory physiology may be even more pronounced in the normal-tension compared with the high-tension form of POA (49).

### Microgravity Effects on the Brain in Space Flight

Although acute exposure to microgravity has immediate physiologic effects, it is the chronic exposure to microgravity that poses a more insidious consequence, as experimented during space flight. The longer the mission lasts the higher is the evidence of visual acuity changes in astronauts associated with structural abnormalities of the retina and optic nerve. The etiology of the ocular changes remains speculative; however, MRI abnormalities identified in postflight astronauts have implicated ICP as the underpinning cause. Intracranial hypertension can hypothetically affect the visual pathway through propagation of elevated CSF pressure from the intracranial compartment to the orbit with resultant expansion of the optic nerve sheath and structural modification of the posterior globe. Kramer et al. (50) verified that astronauts with radiological sign of intracranial hypertension in the postflight had significant lower CSF production in the preflight. Therefore, it was considered that those subjects had a higher baseline CSF outflow resistance and/or sagittal sinus pressure (50). Summarizing, these physiologic changes occurring during exposure to microgravity may help elucidate mechanisms responsible for terrestrial IIH.

### Complementary Tests of the Hypothesis

MRI studies provide indirect evidence of cerebral edema, associated with clinical data on the swollen brain (papillary edema, headache, and increased CSF pressure) (28). This set of signs and symptoms motivate us to question whether cerebral clearance (glymphatic system) would cause slowing and stagnation in the brain microcirculation leading to IF accumulation.

Given the high sensibility of the empty sella for a diagnosis of IIH and the fact that the pituitary recess is one of the most proximal glymphatic inflow conduits, the sellar glymphatic compartment is probably affected early in the course of IIH and represents an area of interest for study (8). Moreover, the diffusion tensor imaging proved to be useful in the evaluation of glymphatic dysfunction in patients with AD (39).

The AQP4 has also been in the focus of HII research. Kerty et al. (23) searched for possible single-nucleotide polymorphisms in the AQP4 gene. Despite the absence of significant polymorphisms, they reason that the examination of polymorphisms alone do not comprise information about the control of gene expression or the post-transcriptional regulation. They advocate the assessment of AQP4 expression and distribution in tissues resected from patients with IIH (23). A radiotracer capable of combining to AQP4 might demonstrate this distribution.

Considering the existence of a possible glymphatic dysfunction associated with slower CSF circulation and reduced CSF production, it would be interesting to verify whether IIH patients present higher levels of CSF protein and other cumulative metabolites (e.g., lactate) than controls. The LP aiming to relieve ICP might unblock stagnated CSF flow: if the restraint or resistance is in the interstitium → venous paravascular direction, as in NPH, significant rises of waste products might be noted comparing CSF composition before and after this procedure. However, if there is an overload in arterial paravascular → interstitium direction, the removal of CSF might not interfere with the interstitium drainage. In the experiment by Lundgaard et al. (16) using microdialysis samples, anesthetized mice without glymphatic dysfunction showed a drop in brain lactate in comparison with the awake state. This drop was prevented by a previous cisterna magna puncture, an effect that was credited to the reduction of the pressure differences that drive CSF exchange with ISF (16). Margeta et al. (51) found significantly lower CSF protein levels in their cohort of prepubertal children with IIH compared with pubertal patients with IIH and controls. The authors hypothesized that the lower CSF protein levels are a result of CSF overproduction. They suggested that protein levels decrease as a function of the increased clearance that follows the increase in CSF production. It is interesting that older children had higher levels of CSF protein, which might suggest a progressive saturation of the exit route or a congestion that hampers the circulation of IF (51).

The concentration of Aβ is another candidate of cumulative metabolite to be checked. Current medicines used to treat IIH, such as acetazolamide, while hampering the production of CSF enhances the acidification in brain, which might lead to the formation of fibrillary Aβ degradation. However, the analysis of this product may have a low sensibility since patients with IIH tend to be young, and the glymphatic flow is disturbed. In this case, a prospective study of IIH might elucidate whether it contributes any risk to the development of AD.

## Discussion

The discovery of the glymphatic system may bear important implications for the diagnosis and management of several common neurological diseases in the near future. For instance, it has been proposed that a failure in the clearance of soluble β-amyloid from the brain interstitium contributes to amyloid plaque deposition and AD progression (2, 8).

Idiopathic intracranial hypertension is responsible for severe headache, papilledema, and visual loss that may evolve into irreversible brain damage. The incidence of IIH is expected to rise given the worldwide obesity epidemic and the well-known predisposing role of obesity for the development of IIH (20). The role of inflammation is supported by Denniston et al.’s study (11), who found an increase in the permeability of the optic nerves in ICP leading to “paravascular black holes.” Ocular perivascular glymphatic channels might in turn explain the delayed visual dysfunction of chronic papilledema in IIH (11).

This article discusses the role of IF accumulation as a consequence of glymphatic dysfunction leading to increased ICP in IIH. MRI signs indicative of IIH have not included direct evidence of overproduction of CSF. Indeed, there seems to occur the clearance saturation of the glymphatic system, whether by a primary disturbance (e.g., AQP4 deficiency) or, for example, by an increase in volume of the PVSs, which could take place in inflammatory conditions such as obesity.

Regardless of the exact mechanism at play in individual cases, we argue that glymphatic dysfunction is a final common pathway for abnormal increases of ICP. Currently, increases of ICP are treated with (a) measures to reduce the total volume of CSF by direct removal either from the SAS through cisternal or LP, (b) by attempts to inhibit CSF secretion at the choroid plexus, or a combination of (a) and (b). The second strategy is provided by drugs, such as acetazolamide and topiramate, which inhibit carbonic anhydrase. It is known that one of the main determinants of CSF secretion is the production of $\text{HC}{\text{O}}_{3}^{-}$ by carbonic anhydrase in the choroid epithelial cells (7). These measures are palliative only, since they probably do not modify the fundamental pathophysiological mechanisms. Moreover, acetazolamide and topiramate are often poorly tolerated for long periods (20). If glymphatic dysfunction lies at the root of IIH, the use of acetazolamide might further hinder the clearance of toxic metabolites. If, as hypothesized in this article, the impairment of CSF circulation in IIH is secondary to a dysfunctional glymphatic system, research should focus on measures that increase the flow between the CSF, the glymphatic system and the dural sinuses.

## Conclusion

The discovery of the glymphatic system may bring novel insights into the pathogenesis of IIH. Confirmatory evidence that IIH is associated with CSF circulatory dysfunction and stagnation in the cerebral microcirculation should redirect the attention of clinicians and researchers to new therapeutic measures aiming at enhancing the rate of cerebral clearance. The present hypothesis affords a direct clinical application of the glymphatic concept. As such, it may open the way for new studies on the role of the glymphatic system in several common neurological diseases, such as AD and other degenerative diseases.

## Author Contributions

MB is the mentor of the central idea explored in the article. He also contributed to the writing of the manuscript. AF contributed in the writing of the manuscript and researched for more arguments to defend the presented hypothesis. RO-S searched for more papers to illustrate the cited arguments. He contributed a great deal to the edition and the improvement of the text.

## Conflict of Interest Statement

The authors declare that the research was conducted in the absence of any commercial or financial relationships that could be construed as a potential conflict of interest.
